# Cannabidiol mitigates alcohol dependence and withdrawal with neuroprotective effects in the basolateral amygdala and striatum

**DOI:** 10.1038/s41386-025-02164-6

**Published:** 2025-07-10

**Authors:** Selen Dirik, Michelle R. Doyle, Courtney P. Wood, Paola Campo, Angelica R. Martinez, McKenzie Fannon, Maria G. Balaguer, Spencer Seely, Bryan A. Montoya, Gregory M. R. Cook, Gabrielle M. Palermo, Junjie Lin, Madelyn D. Sist, Parsa K. Naghshineh, Zihang Lan, Sara R. M. U. Rahman, Raymond Suhandynata, Paul Schweitzer, Marsida Kallupi, Giordano de Guglielmo

**Affiliations:** 1 Department of Psychiatry, University of California San Diego, School of Medicine, La Jolla, CA USA; 2 The Scripps Research Institute, La Jolla, CA USA; 3 Department of Pathology, University of California San Diego, School of Medicine, La Jolla, CA USA; 4 Skaggs School of Pharmacy and Pharmaceutical Sciences, University of California San Diego, School of Medicine, La Jolla, CA USA

**Keywords:** Preclinical research, Addiction

## Abstract

Alcohol use disorder (AUD) remains a pervasive public health issue with limited effective treatments. Cannabidiol (CBD), a non-psychotropic constituent of cannabis, shows promise in modulating addictive behaviors. This study investigated the effects of chronic CBD administration on alcohol dependence, withdrawal symptoms, and neurodegeneration using two complementary rodent models: chronic intermittent ethanol (CIE) exposure, which models established alcohol dependence, and ethanol vapor self-administration (EVSA), which captures the volitional aspects of alcohol intake. In the CIE model, CBD reduced alcohol self-administration during acute withdrawal without affecting alcohol metabolism or locomotor activity. CBD decreased motivation for alcohol, somatic withdrawal signs, withdrawal-induced anxiety-like behaviors, and mechanical sensitivity. During extinction, CBD attenuated alcohol-seeking behavior and stress-induced reinstatement. Electrophysiological recordings revealed that CBD reversed alcohol-induced decreases in neuronal excitability in the basolateral amygdala, suggesting a mechanism involving normalization of neural function. In the EVSA model, CBD reduced voluntary alcohol intake during the escalation phase, impacting voluntary alcohol intake. This effect was specific to alcohol-related behaviors, as it did not affect saccharin self-administration. Immunohistochemical analyses showed that CBD prevented alcohol-induced neurodegeneration in the nucleus accumbens shell and dorsomedial striatum, regions implicated in the volitional control of alcohol consumption. These findings indicate that chronic CBD administration attenuates both behavioral and neurobiological facets of alcohol dependence by modulating neuronal excitability and preventing neurodegeneration, supporting its therapeutic potential for AUD and providing mechanistic insights for future research.

## Introduction

Alcohol use disorder (AUD) is a prevalent and debilitating condition characterized by compulsive alcohol consumption, loss of control over intake, and a negative emotional state during withdrawal [1]. Despite the significant health, social, and economic burdens posed by AUD, current pharmacotherapies are limited by modest efficacy and undesirable side effects [2–5]. Less than 10% of individuals with AUD receive approved medications [6], highlighting the urgent need for novel therapeutic approaches [7].

Cannabidiol (CBD), a non-psychotropic constituent of the *Cannabis sativa* plant, has garnered considerable interest for its potential therapeutic properties across a range of neuropsychiatric disorders [8, 9]. Unlike delta-9-tetrahydrocannabinol (THC), CBD is not intoxicating and has demonstrated a favorable safety and tolerability profile [9, 10]. CBD exhibits a diverse pharmacological profile, including neuroprotective, anti-inflammatory, anxiolytic, and anticonvulsant properties [11, 12], making it a promising candidate for the treatment of AUD.

Preclinical studies have shown that CBD can reduce alcohol consumption and attenuate alcohol-seeking behaviors in rodent models [13–17], suggesting that CBD may modulate the reinforcing properties of alcohol and reduce the risk of relapse. CBD has also demonstrated neuroprotective effects against alcohol-induced neurodegeneration. Chronic alcohol consumption leads to neuronal cell death and cognitive deficits, particularly in brain regions such as the hippocampus and entorhinal cortex [18]. CBD was found to protect against alcohol-induced neurotoxicity in these regions by reducing oxidative stress and inflammation in preclinical models [19–21]. Additionally, CBD prevented alcohol-induced hepatotoxicity and steatosis in mice, potentially through its anti-inflammatory and antioxidant properties [22, 23]. These neuroprotective effects may contribute to the mitigation of cognitive and behavioral impairments associated with AUD [24].

Despite the promising preclinical evidence supporting CBD’s potential therapeutic effects, most studies to date have evaluated CBD in non-dependent animals using limited exposure paradigms. A thorough evaluation of CBD’s therapeutic potential for AUD requires investigation using validated models of alcohol dependence that better reflect the human condition. To address this critical gap, the present study investigated the effects of chronic CBD administration on alcohol dependence, withdrawal symptoms, and neurodegeneration using two complementary rodent models. The chronic intermittent ethanol (CIE) exposure model induces physical dependence through passive exposure to ethanol vapor, mimicking neuroadaptive changes and withdrawal symptoms observed in humans [25–30]. This model allows for examination of neural substrates associated with established alcohol dependence. In contrast, the ethanol vapor self-administration (EVSA) model captures the volitional aspects of alcohol intake and the transition to dependence by allowing animals to voluntarily self-administer ethanol vapor, thereby modeling the development of habitual excessive drinking behavior [31, 32].

Here, we aimed to elucidate the mechanisms by which chronic CBD treatment attenuates alcohol dependence and its associated neurobiological alterations. We hypothesized that CBD would reduce alcohol intake, alleviate withdrawal symptoms, and prevent alcohol-induced neurodegeneration by modulating neuronal excitability and normalizing neural function in key brain regions.

## Materials and methods

### Subjects

Adult Wistar rats (n = 166 total; 87 males, 79 females) were obtained from Charles River. Experiments began when rats were 10-12 weeks old. Rats had access to water and standard laboratory chow (PJ Noyes Company) *ad libitum* in their home cage. Rats were housed in a temperature- (20–22°C) and humidity-controlled (45–55%) environment on a 12 h/12 h reverse light/dark cycle, with lights on at 9 p.m. All the procedures adhered to the National Research Council’s Guide for the Care and Use of Laboratory Animals and were approved by the Institutional Animal Care and Use Committee of the University of California, San Diego.

### Drugs

Synthetic cannabidiol (Purisys LLC) was dissolved in sesame oil (Sigma) at doses of 0, 30, and 60 mg/kg and injected subcutaneously (SC) 30 minutes prior to the tests. These doses were selected based on previous studies demonstrating CBD’s efficacy in preclinical rodent models [33–36] and its favorable safety profile at these concentrations [10]. Yohimbine hydrochloride (Sigma) was dissolved in sterile water and administered intraperitoneally (IP) at 1.25 mg/kg 30 minutes prior to the test [37].

## Experiment 1: Effects of chronic CBD treatment on alcohol dependence

### Alcohol self-administration training and escalation

Self-administration occurred in operant chambers (Med Associates) where the right panel of the chamber was equipped with two retractable levers and two sipper cups. Rats (n = 32; 16/sex, divided into two cohorts of 16 rats for 30 mg/kg and 60 mg/kg doses) were trained to self-administer water (0.1 ml/reinforcer) in a 16-hour overnight FR1 session, followed by a 16-hour ethanol (10% v/v, 0.1 ml/reinforcer) session with *ad libitum* access to food but no other fluid sources. Rats then completed three 30-min FR1 ethanol sessions before transitioning to concurrent ethanol (right lever) and water (left lever) access in daily 30-min FR1 sessions. This non-dependent phase ran for 14 daily sessions (Monday-Friday).

### Chronic intermittent ethanol exposure

After establishing stable baseline responding (>3 weeks), rats underwent chronic intermittent ethanol (CIE) vapor exposure for 3 weeks to induce dependence. They were housed in vapor chambers for 14 hours daily, with vapor levels maintaining blood alcohol levels (BALs) at 150-225 mg/dl, monitored weekly via tail vein sampling and gas chromatography [32]. Self-administration resumed on Mondays, Wednesdays, and Fridays during acute withdrawal (6-8 hours post-vapor).

### CBD treatment and alcohol self-administration

Following establishment of dependence, rats were randomly assigned to receive daily subcutaneous injections of either vehicle (sesame oil) or CBD (30 mg/kg, or 60 mg/kg) for four weeks, administered 30 minutes prior to the behavioral sessions or at the same time of day on non-session days. Two separate studies, each with distinct rat cohorts, evaluated the effects of CBD 30 mg/kg (Study 1) or CBD 60 mg/kg (Study 2) versus vehicle on alcohol self-administration. Each study assessed outcomes over eight sessions during acute withdrawal (6–8 hours post-vapor termination), with data analyzed independently.

### Progressive ratio testing

After 8 self-administration sessions during CIE exposure, motivation for alcohol was assessed using a progressive ratio (PR) schedule, where the response requirement increased according to the sequence: 1,1,2,2,3,3,4,4,5,5,7,7,9,9,11,11 [31, 38]. The breakpoint was defined as the final completed ratio before a 45-minute period without completion of the required responses. CBD (60 mg/kg, SC) was administered 30 minutes before testing.

### Alcohol metabolism

To evaluate CBD’s impact on alcohol metabolism, a separate cohort of Wistar rats (n = 24, 12 per sex) was used. Blood samples were collected via tail vein every hour for 6 hours following vapor termination in rats receiving chronic CBD treatment (60 mg/kg, 4 weeks). Blood alcohol concentrations were analyzed using gas chromatography.

### Behavioral assessments during acute withdrawal

All behavioral assessments were conducted on the same cohort of rats from section “CBD Treatment and Alcohol Self-Administration” (n = 16, 8/sex), treated with daily subcutaneous injections of CBD (60 mg/kg) or vehicle (sesame oil) 30 minutes before behavioral sessions or at the same time on non-session days. Alcohol intake was measured on Mondays, Wednesdays, and Fridays, while behavioral tests (locomotor activity, somatic withdrawal signs, anxiety-like behavior, and mechanical sensitivity) were performed on Tuesdays and Thursdays during acute withdrawal (6–8 hours post-vapor termination).

### Locomotor activity

Locomotor activity was assessed during acute withdrawal (6-8 hours post-vapor) after vehicle or CBD (60 mg/kg, SC). The open field, a roofless black plexiglass square (50 ×50 x 40 cm), was placed on the floor. Rats started in the bottom left corner, and activity was recorded for 15 minutes using AnyMaze under standardized conditions.

### Somatic withdrawal signs

Withdrawal severity was assessed using a scale adapted from Macey et al. [39], rating ventromedial limb retraction (VLR), abnormal gait, vocalization, tail stiffness, and tremors (0-2: none to severe; total 0-10). Evaluations occurred during acute withdrawal after vehicle or CBD (60 mg/kg, SC), conducted by a blinded experimenter.

### Anxiety-like behavior

Anxiety-like behavior was evaluated using an elevated plus maze constructed of black plastic (two open arms, 50×10 cm; two closed arms with sidewalls; 50 cm high). During 5-minute sessions under dim red light, a blinded observer recorded arm entries and time spent in each arm following administration of vehicle or CBD (60 mg/kg, SC) during acute withdrawal.

### Mechanical sensitivity

Mechanical nociception was assessed with a dynamic plantar aesthesiometer (Ugo Basile) during acute withdrawal. Three force measurements (g) for hind paw withdrawal were recorded and expressed as absolute values post-CBD (60 mg/kg, SC) or vehicle.

## Experiment 2: CBD blood levels and control studies for non-specific effects

In this set of experiments, we aimed to establish the plasma levels of CBD after an injection of 60 mg/kg SC and performed control experiments to demonstrate CBD does not potentiate the sedative effects of alcohol.

### CBD plasma levels

Adult Wistar rats (n = 8; 6 males, 2 females), housed under conditions described in the Subjects section, received a single subcutaneous injection of synthetic cannabidiol (CBD; 60 mg/kg, dissolved in sesame oil). Thirty minutes post-injection, blood was collected via tail vein puncture. Plasma CBD concentrations were determined from 50 μL of plasma (collected with K-EDTA as the anticoagulant) using isotope dilution mass spectrometry (100 ng/mL D3-cannabidiol), as previously described [40].

### Effect of CBD on alcohol-induced loss of righting reflex (LORR)

Adult Wistar rats (n = 16; 12 males, 4 females), housed as described in the Subjects section, were randomly assigned to receive a subcutaneous injection of CBD (60 mg/kg, dissolved in sesame oil; n = 8) or vehicle (sesame oil; n = 8). Thirty minutes later, rats received an intraperitoneal injection of ethanol (2.5 g/kg, 20% v/v in saline). Immediately post-ethanol injection, each rat was placed in a supine position on a V-shaped platform [41]. The time to loss of righting reflex (LORR; interval from ethanol injection to inability to right itself) and duration of LORR (interval from loss of righting ability to three consecutive successful rightings within 60 seconds) were recorded by a blinded observer.

### Effect of CBD on locomotor activity during alcohol intoxication

The same Wistar rats from Experiment 2.2 (n = 16; 12 males, 4 females) underwent chronic intermittent ethanol (CIE) vapor exposure for 2 weeks to induce dependence, as described in section “CBD Treatment and Alcohol Self-Administration”, with blood alcohol levels maintained at 150–225 mg/dL. Rats received daily subcutaneous injections of CBD (60 mg/kg, dissolved in sesame oil; n = 8) or vehicle (sesame oil; n = 8) throughout the 2-week period. On day 15, immediately following the final 14-hour vapor exposure (blood alcohol levels ~200 mg/dL) and 30 minutes after the last CBD or vehicle injection, locomotor activity was assessed in an open-field test, as described in section “Locomotor Activity”.

## Experiment 3: Effects of CBD on stress-induced reinstatement of alcohol seeking

A separate cohort of rats (n = 24; 12/sex) underwent alcohol self-administration training and CIE exposure as in Experiment 1. The pre-vapor baseline was calculated as the average alcohol intake (g/kg) from the last three 30-minute self-administration sessions before CIE exposure. After 3 weeks of CIE, self-administration resumed during acute withdrawal (6–8 hours post-vapor termination) on Mondays, Wednesdays, and Fridays for 2–3 weeks until escalation occurred, defined as a significant increase (p < 0.05, paired t-test) in the average intake from the final three sessions compared to the pre-vapor baseline. Post-dependence, rats were removed from vapor exposure and randomized to vehicle (sesame oil) or CBD (60 mg/kg, SC; n = 12/group) treatment during extinction.

### Extinction training

Post-alcohol self-administration, rats underwent daily 30-minute extinction sessions under training conditions, but without alcohol (lever responses had no consequences). Sessions ran for two weeks where daily CBD (60 mg/kg, SC) or vehicle was administered 30 min before every session.

### Yohimbine-induced reinstatement

Twenty-four hours after the final extinction session, rats were tested for stress-induced reinstatement. CBD (60 mg/kg, SC) or vehicle was given 60 min before the session, followed by yohimbine (an α2-adrenergic receptor antagonist that induces stress-like responses) [42], 30 min prior [37]. During the 30-minute test, extinction conditions applied, lever responses were recorded but had no consequences.

## Experiment 4: Effects of CBD on alcohol-induced changes in BLA neuronal excitability

### Animals and treatment

Wistar rats (n = 12; 6/sex) underwent alcohol self-administration and CIE exposure until escalation occurred (following the procedures of Experiment 1 and 3). After the escalation of intake was achieved, rats underwent 2-week abstinence with daily CBD (60 mg/kg, SC) or vehicle injections. Naïve control rats (n = 6; 3/sex), served as a comparison group. The 2-week abstinence period with daily CBD treatments was chosen to align with the extinction protocol in Experiment 2, ensuring consistent chronic treatment and abstinence conditions for parallel evaluation of CBD’s effects on relapse-like behavior and BLA excitability. The BLA was chosen for its role in withdrawal/relapse [43–45].

### Brain slice preparation

Thirty minutes after the final CBD or vehicle injection, rats were euthanized, and brains were rapidly extracted and placed in ice-cold sucrose solution containing (in mM): 206.0 sucrose, 2.5 KCl, 0.5 CaCl_2_, 7.0 MgCl_2_, 1.2 NaH_2_PO_4_, 26 NaHCO_3_, 5.0 glucose, and 5 HEPES. Coronal slices (300 μm thick) containing the BLA were prepared using a Leica VT1200S microtome. Slices were incubated in oxygenated (95% O_2_/5% CO_2_) artificial cerebrospinal fluid (aCSF) containing (in mM): 120 NaCl, 2.5 KCL, 5 EGTA, 2.0 CaCl2, 1.0 MgCl_2_, 1.2 NaH_2_PO_4_, 26 NaHCO_3_, 1.75 glucose, and 5 HEPES. Slices were maintained at 37 °C for 30 minutes followed by 30-minute equilibration at room temperature (20-22 °C).

### Electrophysiological recordings

Whole-cell current-clamp recordings from BLA neurons used borosilicate pipettes (3-5 MΩ) filled with KCl-based internal solution (pH 7.2-7.4, 285-295 mOsm). Recordings were made with a MultiClamp 700B amplifier, filtered at 2 kHz, digitized at 10 kHz, and analyzed using pClamp 10.7 (Molecular Devices). Neurons were visualized with an Olympus BX51WI microscope under infrared differential interference contrast. Inclusion criteria were resting membrane potential < -50 mV and action potential amplitude > 60 mV; cells with >20% series resistance change were excluded. Intrinsic properties (resting potential, input resistance) were measured in current-clamp mode, with input resistance derived from -100 pA steps (500 ms). Excitability was assessed via action potentials evoked by 500-ms current injections (0 to +200 pA, 25 pA steps), counted for input-output curves. Recordings occurred at 20-22°C in oxygenated aCSF (2-3 ml/min).

## Experiment 5: Effects of CBD on voluntary alcohol vapor self-administration and saccharin self-administration

### Ethanol vapor self-administration (EVSA)

#### Apparatus

The EVSA apparatus consisted of modified rat home cages equipped with two nosepoke holes and corresponding cue lights for active and inactive responses [31, 32]. Each chamber was connected to a custom alcohol vaporization system comprising a heating element, glass flask, two solenoids (one “normally opened” for clean air, one “normally closed” for alcohol), a gas washing bottle, and a compressor. The system was controlled by a Med Associates smartcard, with a minimum ventilation rate of 15 L/min clean air throughout experiments.

#### EVSA training and testing

Male and female Wistar rats (n = 32, 16 per sex) were randomly assigned to receive either CBD (60 mg/kg, SC; n = 16) or vehicle (n = 16) 1 hour before each test session. Animals underwent 8-hour sessions every other day (10 AM to 6 PM) for 26 total sessions, with food and water withheld during testing. The protocol consisted of three phases [31, 32]:

- Sessions 1-8: Each active nosepoke triggered 2-minute alcohol vapor exposure (15 L/min) paired with a 20-second cue light (timeout period). Responses during timeout or in the inactive nosepoke hole had no programmed consequences.
- Sessions 9-16: Parameters remained identical except vapor exposure duration increased to 5 minutes per response.
- Sessions 17-26: Vapor exposure duration increased to 10 minutes per response.

### Saccharin self-administration control

#### Apparatus and procedure

A separate group of Wistar rats (n = 20, 10 per sex) was trained to self-administer saccharin solution (0.04% w/v in tap water; 0.1 ml/reinforcer) using standard operant chambers as described in Experiment 1. Animals had concurrent access to saccharin (right lever) and water (left lever) on an FR1 schedule. CBD (60 mg/kg, SC) or vehicle was administered 1 hour before each session and followed the same treatment protocol as the EVSA experiment.

## Experiment 6: Effects of CBD on alcohol-induced neurodegeneration in striatal subregions

Following the EVSA protocol (Experiment 4), neurodegeneration markers were assessed in the NAc shell, core, dorsomedial (DMS), and dorsolateral striatum (DLS). The striatum was selected for neurodegeneration markers in the EVSA model for its role in volitional dependence [32]. Rats were euthanized by CO_2_ overdose 1-hour post-final CBD (60 mg/kg) or vehicle injection, perfused with 150 mL ice-cold saline and 400 mL 4% paraformaldehyde (PFA), and brains post-fixed in 4% PFA overnight at 4 °C. After cryoprotection in 30% sucrose with 0.1% sodium azide in PBS, 40 µM coronal sections were cut via cryostat and mounted.

For immunohistochemistry, sections underwent antigen retrieval (10 mM citrate buffer, pH 6.5), peroxidase quenching (1% H₂O₂ in PBS), and blocking with 5% serum (horse for NeuN; goat for others) plus 0.5% Triton X-100. Primary antibodies included anti-NeuN (MAB377, Millipore Sigma; 1:1000), NG2 (PA5-100235, ThermoFisher; 1:200), Cleaved Caspase-3 (9661, Cell Signaling; 1:500), and anti-GFAP (Z0334, Agilent; 1:500). Biotinylated secondary antibodies (Horse anti-Mouse for NeuN; Goat anti-Rabbit for others, Vector Labs) preceded visualization with VECTASTAIN Elite ABC and DAB kits (Vector Labs). Sections were dehydrated (95% ethanol, 100% ethanol, CitriSolv) and coverslipped with DPX Mountant (Sigma-Aldrich).

Images were captured using a Keyence VHX-X1 microscope at 10X (Caspase-3, NeuN) or 20X (GFAP, NG2). Three sections per region (NAc shell, core, DMS, DLS) were analyzed with ImageJ (NIH) by a blinded observer, counting immunoreactive cells via Particle Analysis in fixed areas, averaged as cells/mm².

### Statistical analysis

Statistical analyses were conducted using GraphPad Prism 9.0. Data are reported as mean ± SEM unless noted. Normality and variance homogeneity were confirmed before parametric tests. For time course data in alcohol self-administration and ethanol vapor self-administration (EVSA), two-way repeated measures ANOVA was used with time and treatment as factors. For comparisons of average responses or measures across conditions (e.g., pre- and post-vapor drinking, baseline matching for extinction and reinstatement), two-way ANOVA was employed. Single measure comparisons between two groups used unpaired t-tests (e.g., progressive ratio, locomotor activity, elevated plus maze, von Frey). Blood alcohol levels were analyzed with two-way ANOVA (treatment and timepoint factors). Somatic withdrawal signs, lacking normality, were evaluated with Mann-Whitney U tests. For electrophysiological data, single measures (e.g., input resistance, resting potential) used one-way ANOVA, while input-output curves used two-way repeated measures ANOVA (current and treatment factors). Immunohistochemistry (IHC) data were analyzed with two-way ANOVA (drug and treatment factors). When significant interactions were found, post-hoc tests such as Holm-Sidak, Bonferroni, or Tukey’s were conducted as appropriate. Detailed statistical methods and results are provided in the figure legends. Significance was set at p < 0.05.

## Results

### Experiment 1: Effects of chronic CBD treatment on alcohol dependence

**Fig. 1:**
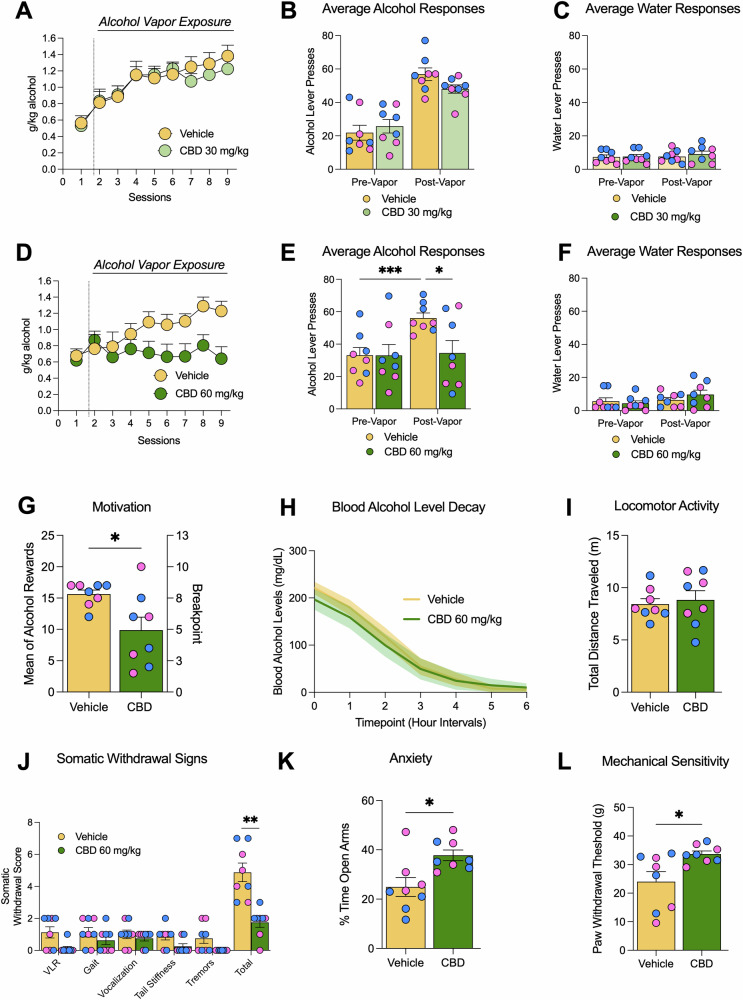
Effects of CBD on alcohol-related behaviors in alcohol dependent rats. **A** Time course of alcohol self-administration with CBD (30 mg/kg; green) or vehicle (yellow) during CIE exposure; two-way repeated measures ANOVA: significant time effect (F(5.248, 73.48) = 11.02, p < 0.001), no treatment or interaction effects. **B** Average responses in the alcohol-paired lever in the last 3 days pre- and post-vapor with CBD (30 mg/kg); two-way ANOVA: significant vapor effect (F(1,15) = 66.68, p < 0.0001). **C** Average responses in the water-paired lever in the last 3 days pre- and post-vapor with CBD (30 mg/kg). **D** Time course with CBD (60 mg/kg); two-way repeated measures ANOVA: significant time × treatment interaction (F(8,112) = 2.301, p = 0.0254). **E** Average responses in the alcohol-paired lever in the last 3 days pre- and post-vapor with CBD (60 mg/kg); two-way ANOVA: significant vapor × treatment interaction (F(1,14) = 7.198, p = 0.0178), Holm-Sidak post-hoc: vehicle escalation (p = 0.0003 vs. pre-vapor), blocked by CBD (p = 0.0059 vs. vehicle). **F** Average responses in the water-paired lever in the last 3 days pre- and post-vapor with CBD (60 mg/kg). **G** Progressive ratio breakpoint; unpaired t-test: t = 2.635, df = 14, p = 0.0196. **H** Blood alcohol levels; two-way ANOVA: no treatment (F(1,21) = 0.1407, p = 0.7114) or interaction effects (F(6,126) = 1.558, p = 0.1649), AUC: 450.1 ± 122.4 (CBD) and 501.3 ± 85.26 (vehicle). **I** Locomotor activity; unpaired t-test: t = 0.3923, df = 14, p = 0.7008. **J** Total somatic withdrawal signs; Mann-Whitney U = 1.000, p < 0.01; individual signs: Ventromedial Limb Retraction (VLR): U = 14.00, p = 0.133; tail stiffness: U = 15.00, p = 0.152; tremors: U = 16.00, p = 0.152 not significant. **K** Time in open arms (elevated plus maze); unpaired t-test: t = 2.949, df = 14, p = 0.0106. **L** Mechanical threshold (von Frey); unpaired t-test: t = 2.632, df = 14, p = 0.0197. Individual data points for male (blue circles) and female (pink circles) rats are shown. Data as mean ± SEM. *p < 0.05, **p < 0.01, ***p < 0.001.

Chronic CBD (30 mg/kg) did not alter the escalation of alcohol self-administration during withdrawal from chronic intermittent ethanol (CIE) vapor exposure, though intake increased over time in both vehicle- and CBD-treated rats (Fig. 1A). Comparing pre- and post-vapor lever presses averages confirmed CIE-induced escalation, unaffected by CBD (Fig. 1B). Average responding for the water-paired lever was low and unaltered by the treatment (Fig. 1C). At 60 mg/kg, CBD showed a differential effect on alcohol intake patterns over time (Fig. 1D) and blocked escalation in post-vapor alcohol-paired lever presses averages, unlike vehicle-treated rats (Fig. 1E). Also for the dose 60 mg/kg, the average responding for the water-paired lever was low and unaltered by the treatment (Fig. 1F) .CBD (60 mg/kg) also lowered the progressive ratio breakpoint for alcohol self-administration (Fig. 1G) without altering blood alcohol level decay (AUC was 450.1 ± 122.4 for CBD and 501.3 ± 85.26 for vehicle, Fig. 1H) or locomotor activity (Fig. 1I). CBD (60 mg/kg) reduced overall somatic withdrawal severity (Fig. 1J), but differences in individual signs (e.g., ventromedial limb retraction, tail stiffness, tremors) did not reach significance. Additionally, CBD attenuated withdrawal-induced anxiety-like behavior in the elevated plus maze (Fig. 1K) and mechanical sensitivity in the von Frey test (Fig. 1L).

### Experiment 2: CBD blood levels and control studies for non-specific effects

**Fig. 2:**
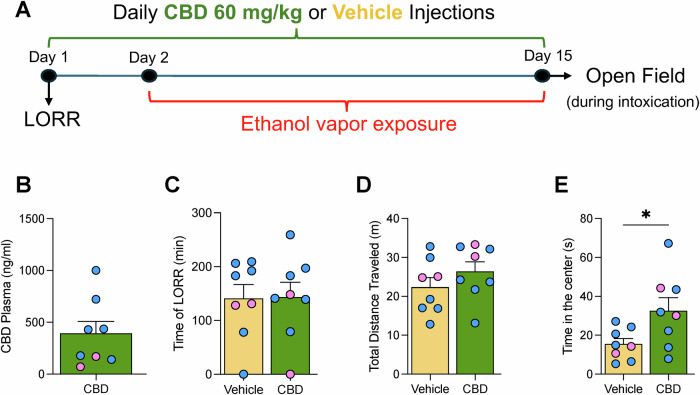
CBD blood levels and effects on alcohol-induced sedation and locomotor activity. **A** Experimental timeline for control studies assessing sedation and locomotor activity. **B** Plasma CBD concentrations (~400 ng/mL) 30 minutes after subcutaneous injection (60 mg/kg) in Wistar rats (n = 8). **C** Loss of righting reflex (LORR) duration following ethanol bolus (2.5 g/kg, i.p.) in CBD (green) and vehicle (yellow) groups; unpaired t-test: t = 1.140, df=7, p = 0.86. **D** Total distance traveled in the open field during alcohol intoxication (blood alcohol levels ~200 mg/dL) after 2 weeks of CIE exposure; unpaired t-test: t = 1.015, df=7, p = 0.98. **E** Time spent in the center of the open field, reflecting anxiety-like behavior; unpaired t-test: t = 5.639, df=7, p = 0.03. Individual data points for male (blue circles) and female (pink circles) rats are shown. Data as mean ± SEM. *p < 0.05.

Plasma CBD concentrations after subcutaneous 60 mg/kg of CBD were approximately 400 ng/mL (Fig. 2B). No difference was observed in loss of righting reflex (LORR) duration between CBD- and vehicle-treated rats (Fig. 2C). Total distance traveled in the open field during alcohol intoxication did not differ between groups (Fig. 2D). However, CBD-treated rats spent significantly more time in the center of the open field, indicative of reduced anxiety-like behavior (Fig. 2E).

### Experiment 3: Effects of CBD on stress-induced reinstatement of alcohol seeking

**Fig. 3:**
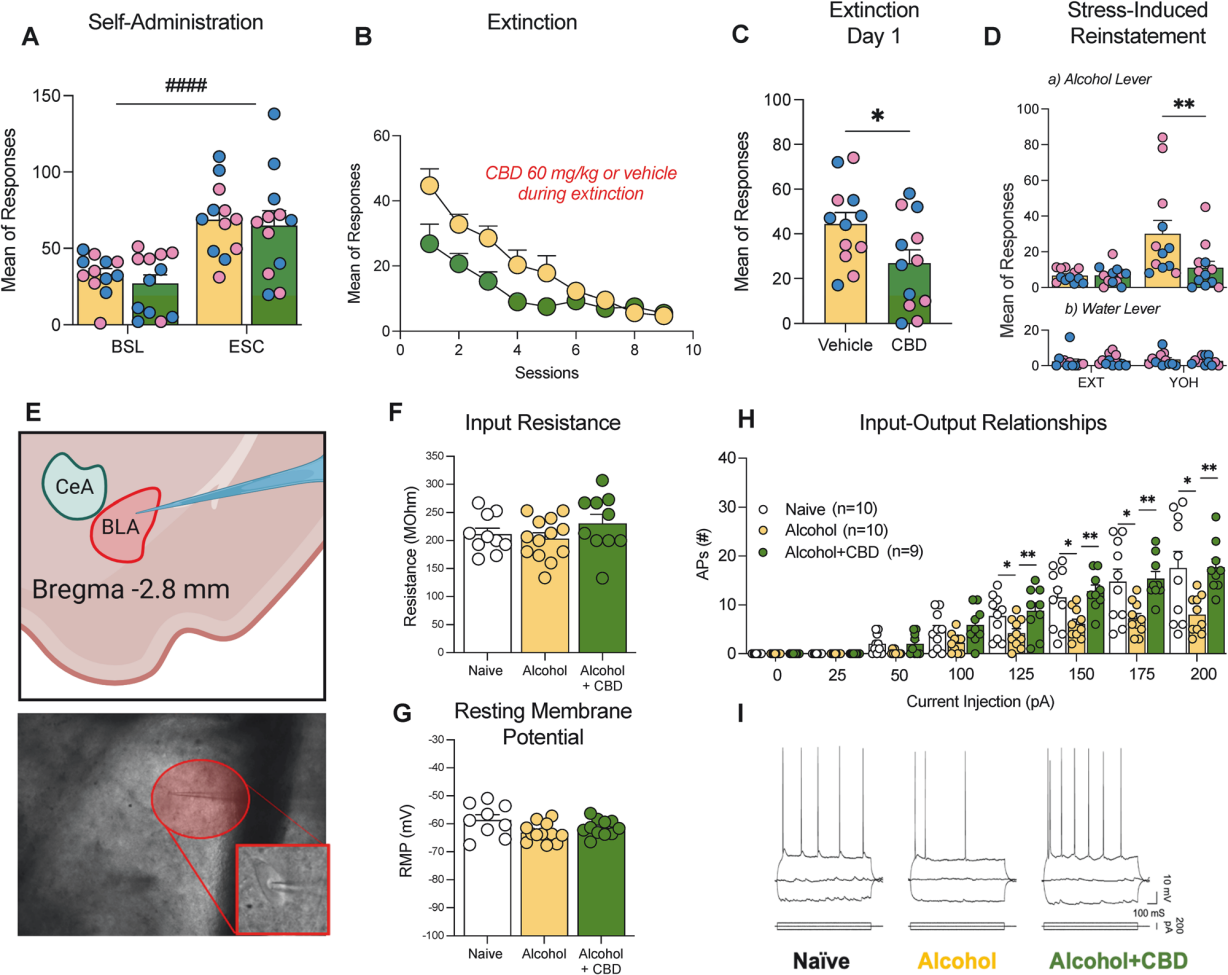
CBD effects on stress-induced reinstatement and on BLA neuronal excitability. **A** Alcohol self-administration pre-treatment; two-way ANOVA: no escalation × group interaction (F(1,22) = 0.02945, p = NS), significant vapor effect (F(1,22) = 34.86, p < 0.0001). **B** Extinction learning curves for CBD (green) and vehicle (yellow); two-way repeated measures ANOVA: significant time × treatment interaction (F(8,176) = 2.795, p = 0.0061). **C** Initial alcohol-seeking on first extinction day; unpaired t-test: t = 2.209, df = 22, p = 0.0379. **D** Yohimbine-induced reinstatement; *panel a)*, Alcohol-paired lever: two-way ANOVA: significant stress × treatment interaction (F(1,22) = 5.651, p = 0.0266), Bonferroni post-hoc: vehicle reinstatement (p = 0.0023), blocked by CBD (p = 0.0069 vs. vehicle). *panel b)*, water-paired lever stress × treatment interaction (F(1,22) = 1.185, p = 0.282). Individual data points for male (blue circles) and female (pink circles) rats are shown. Data as mean ± SEM. *p < 0.05, **p < 0.01, ####p < 0.0001 (main effect). **E** Representative whole-cell current-clamp recordings. **F** Input resistance; one-way ANOVA: F(2,30) = 1.283, p = 0.2874. **G** Resting membrane potential; one-way ANOVA: F(2,29) = 3.159, p = 0.0573. **H** Input-output relationships of action potential firing; two-way ANOVA: significant current × treatment interaction (F(14,182) = 4.373, p < 0.001), Tukey’s post-hoc: alcohol-dependent vs. naive (p < 0.05, ≥125 pA), CBD-treated vs. alcohol-exposed (p < 0.01), CBD comparable to naive. **I** Representative traces of action potential firing. Data as mean ± SEM. *p < 0.05, **p < 0.01.

Animals were assigned to vehicle or CBD groups based on matched pre-treatment alcohol intake, with no baseline differences (Fig. 3A). Both groups escalated alcohol intake post-vapor exposure (Fig. 3A). CBD-treated rats showed reduced alcohol-seeking during extinction, with lower responding from day 1 persisting without a faster decline (Fig. 3B, C). After extinction (<10 lever presses/session), yohimbine testing revealed that CBD blocked stress-induced reinstatement of alcohol seeking, unlike vehicle-treated rats, which showed significant reinstatement (Fig. 3D upper panel). Water-paired lever responses were very low and unaffected by Yohimbine of CBD administration (Fig. 3D lower panel).

### Experiment 4: Effects of CBD on alcohol-induced changes in BLA neuronal excitability

Whole-cell current-clamp recordings assessed BLA neuronal properties after chronic alcohol exposure and CBD treatment (Fig. 3E). Input resistance and resting membrane potential showed no differences across groups (Fig. 3F, G). However, action potential firing in response to depolarizing currents revealed reduced excitability in alcohol-dependent neurons compared to naive controls, an effect reversed by chronic CBD (60 mg/kg) treatment during abstinence, restoring firing rates to naive levels (Fig. 3H, I).

### Experiment 4: Effects of CBD on voluntary alcohol vapor self-administration and saccharin self-administration

**Fig. 4:**
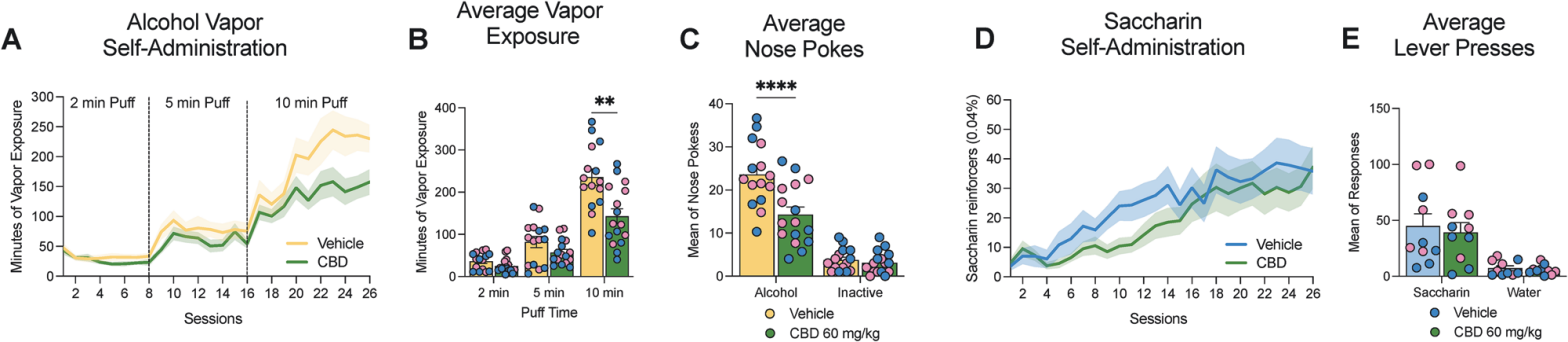
CBD effects on voluntary alcohol vapor and saccharin self-administration. **A** Time course of alcohol vapor self-administration for CBD (green) and vehicle (yellow); two-way repeated measures ANOVA: significant time × treatment interaction (F(25,725) = 2.421, p = 0.0001). **B** Average vapor exposure (2-min, 5-min, 10-min phases); two-way ANOVA: significant exposure time × treatment interaction (F(2,58) = 7.989, p = 0.0009), Holm-Sidak post-hoc: CBD reduced 10-min phase (p = 0.0041 vs. vehicle), no effect at 2-min or 5-min. **C** Average responses in the alcohol-paired and inactive nose pokes for vehicle and CBD treated animals during the last 3 days of the 10 min phase; two-way ANOVA: significant nose-poke × treatment interaction (F(1,29) = 12.78, p = 0.0013), Holm-Sidak post-hoc: CBD reduced nose pokes only in the alcohol-paired side (p = 0.0001 vs. vehicle). **D** Time course of saccharin self-administration for CBD (green) and vehicle (light blue); two-way repeated measures ANOVA: no treatment effect (F(1,18) = 0.9202, p = 0.3501) or time × treatment interaction (F(25,450) = 0.8611, p = 0.6609). **E** Average responses in the saccharin and water paired levers for vehicle and CBD treated animals during the last 3 days of the study. No lever x treatment interaction (F(1,18) = 0.087, p = 0.77), significant main effect of lever (F(1,18) = 25.51, p = 0.0001). Individual data points for male (blue circles) and female (pink circles) rats are shown. Data as mean ± SEM. **p < 0.01.

CBD treatment altered alcohol vapor self-administration patterns across sessions (Fig. 4A). Analysis of average vapor exposure in the last three sessions of each phase (2-min, 5-min, 10-min) showed CBD selectively reduced self-administration during the escalation phase (10-min), but not in earlier phases (Fig. 4B). Inactive nose poke responses remained low and unaffected by the treatment as demonstrated by the average in the last 3 days of the escalation phase shown in Fig. 4C. In contrast, CBD had no effect on saccharin self-administration, indicating specificity to alcohol-motivated behavior rather than general reward-seeking (Fig. 4D). Responses on the water-paired lever in the saccharin experiment were low and unaffected by CBD (Fig. 4E).

### Experiment 5: Effects of CBD on alcohol-induced neurodegeneration in striatal subregions

**Fig. 5:**
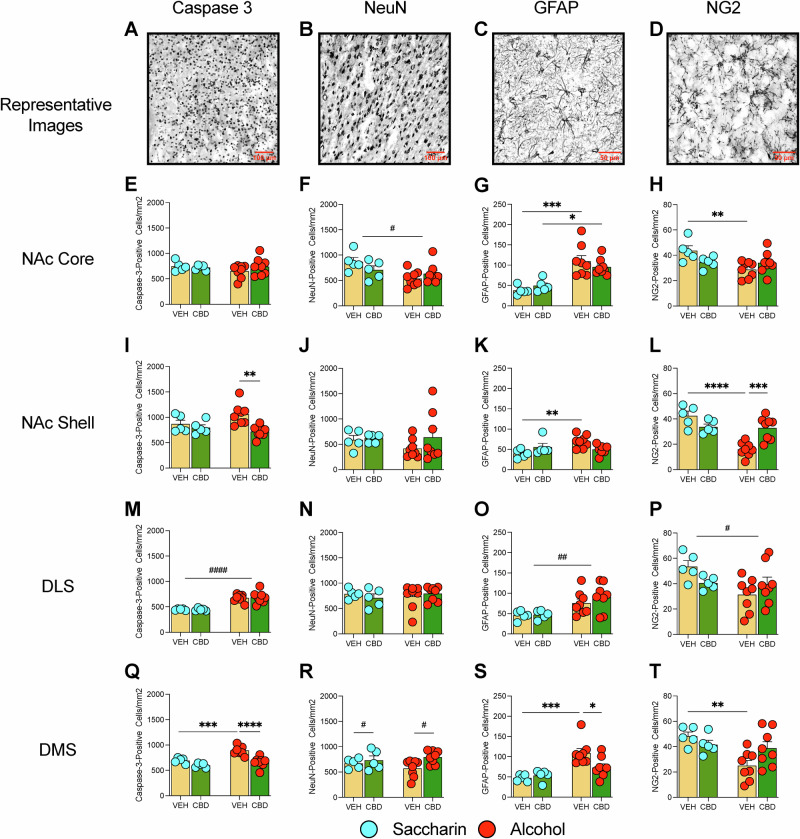
CBD Effects on Striatal Neurodegeneration. Bar graphs show CBD (green) or vehicle (yellow) effects with alcohol (red dots) and saccharin (blue dots) groups. Representative images: **A** Caspase-3 (20X), **B** NeuN (20X), **C** GFAP (40X), **D** NG2 (40X). **NAc Core**: **E** Cleaved caspase-3; two-way ANOVA: no interaction (F(1,22) = 1.169, p = 0.293), treatment (F(1,22) = 0.3967, p = 0.5353), or drug effects (F(1,22) = 0.5148, p = 0.4806). **F** NeuN; drug effect (F(1,22) = 7.928, p = 0.0101), no treatment (F(1,22) = 0.2039, p = 0.6560) or interaction (F(1,22) = 3.229, p = 0.0861). **G** GFAP; drug effect (F(1,22) = 31.91, p < 0.0001), no treatment (F(1,22) = 0.01022, p = 0.9204) or interaction (F(1,22) = 1.525, p = 0.2299). **H** NG2; interaction (F(1,22) = 5.988, p = 0.0229), Tukey’s: alcohol vs. saccharin, p < 0.01. **NAc Shell**: **I** Caspase-3; interaction (F(1,22) = 4.327, p = 0.0494), Tukey’s: CBD vs. vehicle in alcohol group, p < 0.01. **J** NeuN; no effects (interaction: F(1,22) = 0.6155, p = 0.4411; treatment: F(1,22) = 1.040, p = 0.3188; drug: F(1,22) = 0.3708, p = 0.5488). **K** GFAP; interaction (F(1,22) = 8.985, p = 0.0066), Tukey’s: alcohol vs. saccharin, p < 0.01; CBD vs. vehicle in alcohol group, p < 0.01. **L** NG2; interaction (F(1,22) = 19.40, p = 0.0002), Tukey’s: alcohol vs. saccharin, p < 0.0001; CBD vs. vehicle in alcohol group, p < 0.001. **DLS**: **M** Caspase-3; drug effect (F(1,22) = 54.60, p < 0.0001), no treatment (F(1,22) = 0.01839, p = 0.8934) or interaction (F(1,22) = 0.05077, p = 0.8238). **N** NeuN; no effects (interaction: F(1,22) = 0.8354, p = 0.3786; treatment: F(1,22) = 0.03201, p = 0.8596; drug: F(1,22) = 0.06905, p = 0.7952). **O** GFAP; drug effect (F(1,22) = 11.78, p = 0.0024), no interaction (F(1,22) = 0.4119, p = 0.5276) or treatment (F(1,22) = 0.6160, p = 0.4409). **P** NG2; drug effect (F(1,22) = 5.114, p = 0.0340), no interaction (F(1,22) = 4.296, p = 0.0501) or treatment (F(1,22) = 0.2095, p = 0.6517). **DMS**: **Q** Caspase-3; interaction (F(1,22) = 4.434, p = 0.0469), Tukey’s: alcohol vs. saccharin, p < 0.001; CBD vs. vehicle in alcohol group, p < 0.0001; drug effect (F(1,22) = 15.82, p = 0.0006); treatment effect (F(1,22) = 24.87, p < 0.0001). **R** NeuN; treatment effect (F(1,22) = 5.335, p = 0.0307), no interaction (F(1,22) = 1.697, p = 0.2061) or drug effect (F(1,22) = 0.1043, p = 0.7498). **S** GFAP; interaction (F(1,22) = 4.897, p = 0.0376), Tukey’s: alcohol vs. saccharin, p < 0.001; CBD vs. vehicle in alcohol group, p < 0.01; drug effect (F(1,22) = 18.51, p = 0.0003). **T** NG2; interaction (F(1,22) = 5.118, p = 0.0339), Tukey’s: alcohol vs. saccharin, p < 0.01; drug effect (F(1,22) = 8.201, p = 0.0090). Data as mean ± SEM. *p < 0.05, **p < 0.01, ***p < 0.001, ****p < 0.0001 vs. controls; #p < 0.05, ##p < 0.01, ####p < 0.0001 (main effect).

We assessed chronic alcohol vapor self-administration and CBD treatment effects on neurodegeneration markers (cleaved caspase-3, NeuN, GFAP, NG2) across striatal subregions. Chronic alcohol vapor self-administration increased GFAP and reduced NG2 across all striatal subregions (NAc core, NAc shell, DLS, DMS), increased cleaved caspase-3 in DLS and DMS, and reduced NeuN in NAc core compared to saccharin controls (Fig. 5E–T). In the NAc core, CBD had no significant effects on these markers (Fig. 5E–H). In the NAc shell, CBD reduced alcohol-induced increases in GFAP and reductions in NG2 compared to vehicle, with no effects on caspase-3 or NeuN (Fig. 5I–L). In the DLS, CBD had no effects on alcohol-induced changes in caspase-3, GFAP, or NG2, with no NeuN changes (Fig. 5M–P). In the DMS, CBD reduced alcohol-induced increases in caspase-3 and GFAP and reductions in NG2 compared to vehicle, with a treatment effect on NeuN (Fig. 5Q–T).

## Discussion

The present study demonstrates that chronic administration of cannabidiol (CBD) attenuates both behavioral and neurobiological manifestations of alcohol dependence in rodent models. Specifically, CBD reduced alcohol intake and withdrawal symptoms, lowered relapse-like behaviors, normalized neuronal excitability in the basolateral amygdala (BLA), and prevented alcohol-induced neurodegeneration in striatal regions associated with reward and habit formation. Additionally, CBD did not potentiate alcohol’s sedative effects, as shown by no differences in loss of righting reflex duration or locomotor activity during alcohol intoxication, while increasing time spent in the open field’s center, indicating anxiolytic effects. These results underscore CBD’s potential therapeutic utility for alcohol use disorder (AUD) and provide mechanistic insights into its actions.

Chronic intermittent ethanol (CIE) exposure is a well-established model that induces physical dependence and mimics the neuroadaptive changes observed in human AUD, particularly during withdrawal [25–30]. In this study, CBD administration during CIE exposure significantly reduced alcohol intake during acute withdrawal without affecting alcohol metabolism or locomotor activity. This aligns with earlier findings that CBD decreases ethanol intake and motivation to drink in rodent models [13–15]. Importantly, CBD also alleviated withdrawal-associated behaviors, such as somatic signs, anxiety-like behavior, and mechanical sensitivity, which are critical components driving the persistence of alcohol dependence and relapse [46]. CBD-treated rats showed reduced alcohol-seeking during extinction, driven by lower initial responding (Fig. 2B, C), likely reflecting a motivational shift that attenuates cue-induced reinstatement [15]. Notably, human studies suggest CBD enhances extinction consolidation, such as in fear conditioning [47], consistent with reduced cue-reactivity. These findings indicate CBD may support behavioral therapies for AUD by weakening alcohol-cue associations, thus lowering relapse risk.

Stress-induced relapse is another major obstacle in AUD treatment [46]. Here, CBD successfully blocked stress-driven reinstatement of alcohol seeking precipitated by the α2-adrenergic receptor antagonist yohimbine [48]. CBD’s anxiolytic properties, partly mediated via serotonergic 5-HT1A receptors [49], likely underlie this capacity to mitigate stress-induced triggers of relapse. Thus, CBD’s behavioral benefits appear multifaceted, addressing both withdrawal and relapse by dampening stress reactivity.

A pivotal finding of this study is that CBD reversed alcohol-induced decreases in neuronal excitability in the BLA. This region is central to alcohol withdrawal and dependence-related behaviors, and its dysregulation contributes to maladaptive processes that drive relapse [43–45]. Previous studies depicted an increase of BLA neuronal excitability during acute alcohol withdrawal or after shorter CIE regimens [50, 51], an effect believed to implicate glutamatergic hyperactivity and plasticity changes resembling long-term potentiation [52]. However, following our protocol of prolonged ethanol exposure (9 weeks of CIE) and a 2-week abstinence, we observed a reduction of neuronal excitability in BLA neurons. This discrepancy may reflect the duration and intensity of alcohol exposure as well as the time point of assessment.

It is possible that prolonged alcohol exposure triggers compensatory or homeostatic plasticity mechanisms to counteract the initial hyperexcitability. Chronic alcohol use can lead to excitotoxicity due to excessive glutamate release, sometimes resulting in neuronal damage and downregulation of excitability over extended periods [53–55]. Moreover, alcohol has been shown to progressively enhance GABAergic inhibition while suppressing glutamatergic transmission in the central amygdala (CeA) [56–58]. Similar time-dependent shifts have been reported in the medial prefrontal cortex (mPFC), where initial withdrawal can surge excitability, followed by diminished activity during protracted abstinence [59, 60]. By compensating for these maladaptive adaptations, CBD-induced restoration of normal BLA excitability may help prevent the affective disturbances as well as the heightened relapse risk associated with chronic alcohol exposure. Similarly, in a model of temporal lobe epilepsy, CBD was found to restore impaired membrane excitability of hippocampal neurons [61], indicating that CBD may have a general capacity to normalize neuronal function in pathological states.

Unlike traditional forced-exposure paradigms, the ethanol vapor self-administration (EVSA) model incorporates volitional elements of alcohol intake, capturing the transition to dependence through active participation [31, 32]. In the EVSA model, CBD administration during the escalation phase not only suppressed the development of alcohol dependence but also prevented neurodegeneration in the nucleus accumbens (NAc) shell and dorsomedial striatum (DMS). In the EVSA model, CBD’s reduction of neurodegeneration markers may be confounded by lower voluntary alcohol intake. While our findings suggest potential neuroprotective effects, future studies with controlled exposure are needed to isolate CBD’s direct effects. These regions are directly implicated in reward and in the shift from goal-directed to habitual drug-seeking behaviors [62, 63]. Chronic alcohol exposure disrupts these processes by eliciting cellular damage and synaptic alterations[64, 65]. Our findings that CBD confers neuroprotection in these areas are consistent with previous work showing its antioxidant and anti-inflammatory properties against alcohol-induced neuronal damage [9, 12, 19, 20]. Although CBD did not prevent neurodegeneration in the NAc core and dorsolateral striatum (DLS), its selective efficacy in the NAc shell and DMS may reflect differential contributions of each subregion to harm avoidance, habit formation, and reward. The NAc shell and DMS are critically involved in early-stage reward processing and goal-directed behaviors, whereas the NAc core and DLS play a more prominent role in habitual responding [62, 63] These results highlight how CBD’s neuroprotective effects may specifically preserve cortical-striatal circuitry related to volitional control, thereby slowing or preventing the transition from voluntary to compulsive alcohol use [32]. Restored BLA excitability and striatal neuroprotection likely converge via BLA-NAc circuits, which are critical in mediating alcohol addiction-like behaviors [43]

CBD’s broad range of targets likely contributes to its beneficial effects. It can act as a negative allosteric modulator of the CB1 receptor under certain conditions [66] and also interacts with 5-HT1A receptors, GPR55, TRPV1 cation channels, μ- and δ-opioid receptors, and peroxisome proliferator-activated receptor gamma (PPARγ) [11, 67–71] These multifaceted mechanisms may help restore homeostatic neuronal activity in the BLA, limit neurodegeneration in the striatum, and reduce withdrawal- and stress-related behaviors. While CBD at 60 mg/kg may have analgesic and anxiolytic effects [72], it lacks sedation (Figs. 1 and 2) and abuse potential [73], supporting its non-psychoactive profile.

The doses used here (30 and 60 mg/kg in rats) parallel other preclinical studies [33–36]. In addition, plasma CBD concentrations following 60 mg/kg subcutaneous administration in rats (~400 ng/mL; Fig. 2B) align with therapeutic levels in human epilepsy trials, where oral doses of 10–20 mg/kg/day achieve steady-state concentrations of ~100–400 ng/mL [74]. These levels support the translational relevance of our findings, as they approximate human doses (~700–1400 mg/day for a 70 kg person) effective for neuropsychiatric conditions. Consistent with prior work, doubling the dose from 30 mg/kg (~180–220 ng/mL) to 60 mg/kg doubled plasma levels, confirming dose-dependent pharmacokinetics [75]. Observational data in humans link CBD to reduced alcohol use [76, 77] and co-administration with alcohol shows no worsened cognitive impairment in healthy volunteers [78–80]. Additionally, CBD may benefit those with alcohol-related sleep disturbances [81] and a recent study further supports CBD’s potential, reducing anxiety and craving in alcoholics [82]. Ongoing trials (NCT05389930, NCT05860699, NCT06512389, NCT05159830, NCT05613608, NCT04873453, NCT03252756) are testing optimal dosing, duration, and sex differences in AUD treatment efficacy.

Our study included both male and female Wistar rats, but exploratory analyses showed no consistent sex differences in CBD’s therapeutic effects. Limited statistical power, due to sample sizes precluded robust sex-specific analyses. Individual data points for males (blue circles) and females (pink circles) are shown in Figs. 1–5. This is particularly relevant given that AUD exhibits distinct sex-specific patterns in humans, and treatment responses may differ between males and females. Future research should incorporate sex as a biological variable to better evaluate CBD’s efficacy across sexes.

In conclusion, chronic CBD administration mitigates key behavioral and neurobiological features of alcohol dependence by reducing withdrawal symptoms, lowering relapse risk, restoring BLA neuronal excitability, and preventing neurodegeneration in striatal regions. Together, these findings highlight CBD’s capacity to preserve functional integrity in neural circuits underlying emotional regulation, reward processing, and habit formation. Further translational research and clinical trials are warranted both to validate CBD’s therapeutic efficacy in human populations and to optimize dosing strategies for individuals with AUD.

## Data Availability

All data that support the findings of this study are available upon request.
